# Intestinal Gasdermins for regulation of inflammation and tumorigenesis

**DOI:** 10.3389/fimmu.2022.1052111

**Published:** 2022-11-25

**Authors:** Wenbin Gong, Kui Yang, Wei Zhao, Jianbao Zheng, Junhui Yu, Kun Guo, Xuejun Sun

**Affiliations:** ^1^ Department of General Surgery, The First Affiliated Hospital of Xi’an Jiaotong University, Xi’an, Shaanxi, China; ^2^ Department of General Surgery, the First Affiliated Hospital of USTC, Division of Life Sciences and Medicine, University of Science and Technology of China, Hefei, Anhui, China

**Keywords:** Gasdermins, pyroptosis, intestinal inflammation, tumorigenesis, intestinal homeostasis

## Abstract

Gasdermins (GSDMs) protein family express in intestinal epithelial cells or lamina propria immune cells, and play a nonnegligible function during gut homeostasis. With the gradually in-depth investigation of GSDMs protein family, the proteases that cleave GSDMA-E have been identified. Intestinal GSDMs-induced pyroptosis is demonstrated to play a crucial role in the removal of self-danger molecules and clearance of pathogenic organism infection by mediating inflammatory reaction and collapsing the protective niche for pathogens. Simultaneously, excessive pyroptosis leading to the release of cellular contents including inflammatory mediators into the extracellular environment, enhancing the mucosal immune response. GSDMs-driver pyroptosis also participates in a novel inflammatory cell death, PANoptosis, which makes a significant sense to the initiation and progression of gut diseases. Moreover, GSDMs are expressed in healthy intestinal tissue without obvious pyroptosis and inflammation, indicating the potential intrinsic physiological functions of GSDMs that independent of pyroptotic cell death during maintenance of intestinal homeostasis. This review provides an overview of the latest advances in the physiological and pathological properties of GSDMs, including its mediated pyroptosis, related PANoptosis, and inherent functions independent of pyroptosis, with a focus on their roles involved in intestinal inflammation and tumorigenesis.

## 1 Introduction

The intestine represents one of the largest immune organs of the human body mostly because of its surface microscopic architecture (1–3). Majority of immunological processes occur in the mucosa, including the epithelium and the underlying lamina propria (4). In general, the commensal bacteria resident in gut lumen, intestinal epithelium and its secretions (such as mucin and antimicrobial peptides), as well as immune cells in the lamina propria, collaboratively maintain intestinal homeostasis (5–7). Remarkably, there is mounting evidence that the protein family of Gasdermins (GSDMs) play a nonnegligible function under such circumstance.

The GSDMs are a recently characterized family of conserved proteins with diverse functions (8). The nomenclature of Gasdermins is based on their expression pattern along the gastrointestinal tract (gas) and skin (dermis) (9). Human genome encodes six paralogous GSDMs genes: *GSDMA, GSDMB, GSDMC, GSDMD, GSDME* (also known as *DFNA5*) and *DFNB59* (also known as *PJVK*) (9). All proteins of GSDMs family except DFNB59, consist of a conserved N-terminal (NT) and C-terminal (CT) domains that interconnected by a linker region (10). Proteolytic cleavage of GSDMs liberates the NT fragment, which assembles in membrane to form pores and executes lytic cell death, namely pyroptosis (11). However, full-length GSDMs are normally not able to induce such pyroptotic cell death due to the presence of the CT domain, which usually interacts with the NT domain by charge-charge and hydrophobic interfaces to auto-inhibit pore formation function (12). Intestinal homeostasis is frequently disrupted due to the stimulation of endogenous damage-associated molecular patterns (DAMPs) and microorganism-derived pathogen-associated molecular patterns (PAMPs), so as to provoke immune response and lead to tissue damage (13). Intestinal GSDMs-induced pyroptosis plays an essential role in the removal of self-danger molecules and clearance of pathogenic organism infection by mediating inflammatory reaction and collapsing the protective niche for pathogens (14). Simultaneously, excessive pyroptosis results in the release of cellular contents including inflammatory mediators into the extracellular environment (15), which will enhance the immune response and further aggravate intestinal injury. In recent years, the relationship between pyroptosis and cancer has become increasingly prominent, and pyroptosis plays a complex role in tumor progression (16). Furthermore, in addition to GSDMs-NT fragment-induced pyroptosis, emerging evidence indicates GSDMs possess intrinsic physiological functions that independent of pyroptotic cell death in gut. This review provides an overview of the latest advances in the physiological and pathological properties of GSDMs, with a focus on their roles involved in intestinal inflammation and tumorigenesis.

## 2 Expression pattern of Gasdermins in gut

Although GSDMs display unique tissue expression pattern in human, it has been found that GSDMA-E exist in the gastrointestinal tract except DFNB59. GSDMA is expressed in epithelial cells within the gastrointestinal tract while frequently silenced in primary gastric cancers and in gastric cancer cell lines (17, 18). GSDMB is expressed in the epithelium of the digestive tract that can be detected in esophagus, stomach, small intestine, colon and rectum (19). A recent study reveal differential distribution of GSDMB among colonic epithelial cell subtypes, predominantly in colonocytes, crypt top colonocytes, and goblet cells, through the supervised analysis of a large-scale scRNA-seq dataset from health controls and inflammatory bowel disease patients (20). GSDMC is prominently expressed in esophagus, stomach, small intestine, caecum and colon, especially in gut epithelial tissue including enterocytes and goblet cells (21–23). GSDME is also mainly expressed in epithelial cells in the intestine, and relative to normal tissue, GSDME expression in gastric and colorectal cancer is epigenetically suppressed by methylation (11, 24–26). Contrast to other members of the GSDMs family, GSDMD is relatively universally expressed in gastrointestinal tract, not only in epithelium, but also in lamina propria immune cells (especially macrophages and dendritic cells), suggesting that GSDMD may play an indispensable role in intestinal homeostasis (15, 27).

## 3 Molecular mechanisms of Gasdermins cleavage and activation

With the gradually in-depth investigation of GSDMs protein family, the proteases that activate GSDMA-E have been identified. Although the types and cleavage sites of the proteases vary considerably, they share a common mechanism to implement such inflammatory cell death. That is, the GSDMs-NT is unleashed and couples with acidic phospholipids in the inner leaflet of cell membrane to oligomerize into pores with an inner diameter of about 18 nm, which leads to water influx, cell swelling and rapid lytic cell death with the extravasation of cytoplasmic contents (12, 28).

**Figure 1:**
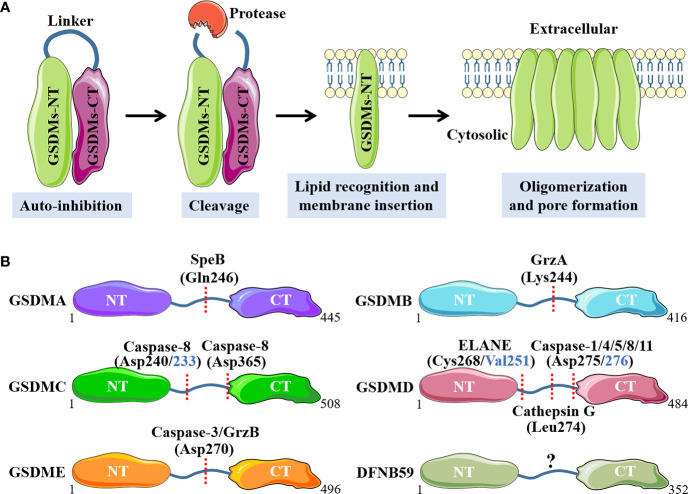
Molecular mechanisms of GSDMs that activated by specific proteases. **(A)**. Main processes of GSDMs membrane pores formation. **(B)**. Respective proteases and cleavage sites for individual GSDMs. NT, N-terminal; CT, C-terminal; SpeB, streptococcal pyrogenic exotoxin B; GrzA, granzyme A; GrzB, granzyme B; ELANE, neutrophil elastase.

Concretely, GSDMA is recently discovered to be cleaved at Gln246 site by cysteine protease streptococcal pyrogenic exotoxin B (SpeB) virulence factor, releasing the active NT fragment that triggers pyroptosis (29). GSDMA acts as not only the sensor and substrate of *Streptococcus pyogenes* SpeB, but also the effector to trigger pyroptosis, adding a novel mechanism for host immune recognition and response to microbial pathogen infection (29). Cytotoxic T lymphocytes (CTL) and natural killer (NK) cells-derived granzyme A (GrzA) cleaves the major Lys244 physiological site of GSDMB to induce pyroptotic cell death (30). Among numerous cytokines, IFN-γ is the one that exhibits broad effects of up-regulation for GSDMB expression in multiple cell lines and promotes GrzA-mediated pyroptosis (30). A previous study has showed that GSDMC is specifically cleaved at Asp365 site by active caspase 8 after TNF-mediated death receptor signaling to trigger pyroptosis (31). Intriguingly, another recently published study has demonstrated that the metabolite α-ketoglutarate (α-KG) induces pyroptosis through active caspase-8-mediated cleavage of GSDMC, but at different amino acid residue site (Asp240, mouse Asp233) (32). GSDMD is the best-characterized GSDMs family protein, and various proteases with capacity to cleave GSDMD have been discovered. Depending on the upstream stimulus signals, when the canonical inflammasomes, including AIM2 sensor by cytosolic double-stranded DNA (dsDNA) or NLRP3 sensor by cellular disturbances and plasma membrane integrity disruption, are activated, GSDMD is cleaved by downstream active caspase-1 at Asp275 site (mouse Asp276) to initiate pyroptosis, accompanied by IL-1 family cytokines IL-1β and IL-18 maturation (33, 34). By comparison, the noncanonical inflammasomes-mediated caspase-4, 5 (mouse caspase-11) activation, also leads to robust cleavage of GSDMD at Asp275 site, but without IL-1 family cytokines production (35). Moreover, in response to pathogenic *Yersinia* infection, where the transforming growth factor beta-activated kinase 1 (TAK1) and IκB kinases are blocked by the *Yersinia* effector protein YopJ, a receptor-interacting protein kinase 1 (RIPK1)-caspase-8 pathway can be activated to cleave GSDMD at this same amino acid site, which provides host defense and maintains homeostasis (36, 37). In addition to the caspases above, granule-associated proteases neutrophil elastase (ELANE) has been shown to cleave and activate GSDMD at Cys268 (mouse Val251) site and cathepsin G cleaves mouse GSDMD directly at Leu274 to provoke pyroptotic cell death (38, 39). GSDME has been shown to be cleaved by active caspase-3 at Asp270 (mouse Asp271) site when stimulated with apoptotic stimuli, which converts apoptosis into a rapid inflammatory pyroptotic death in GSDME-expressing cells (40). In addition, the serine protease granzyme B (GrzB) released from cytotoxic lymphocytes is found to cleave GSDME at the same site to induce pyroptosis (41). Molecular mechanisms of individual GSDMs that activated by specific proteases are summarized in **Figure 1**.

## 4 Function of Gasdermins-mediated pyroptosis in gut homeostasis

When the intestine encounters the attack from self-DAMPs or microbial PAMPs, the mucosal immune system will be initiated, which triggers the immune inflammatory response and destroys the gut homeostasis. Due to the universal existence of GSDMs protein family in intestine, either in epithelium or lamina propria immune cells, its mediated pyroptosis plays a crucial role in the maintenance of abnormal inflammation and clearance of pathogens, including the beneficial and detrimental, depending on the pathological environment at that time. Increasingly emerging evidence indicates that GSDMs-mediated pyroptosis plays an important role in a variety of intestinal diseases, including inflammatory bowel diseases (IBD), sepsis‐induced intestinal injury, enteropathogenic microbial infection, intestinal ischemia/reperfusion (I/R) injury, radiation-induced enteropathy, gastrointestinal dysmotility, colorectal cancer (CRC) and so on.

### 4.1 Gasdermins-mediated pyroptosis in IBD-associated intestinal inflammation

IBD, including Crohn’s disease (CD) and ulcerative colitis (UC), represents a chronic and complex intestinal disorder characterized by uncontrolled and excessive mucosal pathogenic inflammation (42). It is wide recognized that DAMPs released from stressed or dead cells are important pathogenic stimuli for inducing and maintaining abnormal mucosal inflammation during IBD (43). Our recent studies demonstrated that nuclear DAMPs, spliceosome-associated protein 130 (SAP130), released from gut mucosa could induce GSDMD-mediated pyroptosis in lamina propria macrophages through sensing by macrophage-inducible C-type lectin/spleen tyrosine kinase (Mincle/Syk) signaling, which exacerbated mucosal inflammation and provoked intestinal tissue injury during CD (44, 45). We also uncovered that adaptor protein stimulator of interferon genes (STING) regulated intestinal inflammation dependent on GSDMD-related pyroptosis in acute dextran sulfate sodium (DSS)-induced colitis, while STING deficiency alleviated intestinal damage that associated with decreased expression of cleaved form of pyroptosis executive protein GSDMD (46).

A recent study demonstrated that the protein phosphatase, pleckstrin homology domain leucine-rich repeat protein phosphatase 2 (PHLPP2), was downregulated in UC and regulated GSDMD-induced intestinal epithelial cells (IECs) pyroptosis by modulating the NF-κB signaling, and PHLPP2 depletion increased the susceptibility to colitis by inducing dramatic activation of caspase-1/GSDMD in IECs (47). Wang et al. (48) observed that the expression of monocarboxylate transporter 4 (MCT4) was markedly increased in intestinal mucosal tissue of IBD, and overexpression of MCT4 triggered caspase-1/GSDMD-mediated canonical pyroptosis in IECs to aggravate intestinal inflammation through the ERK1/2-NF-kB pathway. CD147, a highly glycosylated transmembrane protein, was recently proved to induce pyroptosis in IECs by enhancing of phosphorylation of NF-κB, which was attributed to activation of caspase-1/GSDMD as well as GSDME, leading to aggravation of IBD (49). It is found that GSDMD in IECs, but not infiltrating immune cells, was activated by dysregulated commensal and in turn modulated microbiota-driven colitis by promoting IL-18 release form pyroptotic cell death (50). Caspase-8 and its adapter Fas associated with death domain (FADD) act on epithelial cells to maintain intestinal immune homeostasis, and FADD prevents intestinal inflammation by inhibiting caspase-8/GSDMD-dependent pyroptosis of IECs (51). In addition, Tan et al. (26) demonstrated that GSDME-mediated IECs pyroptosis induced intestinal inflammation through the release of proinflammatory intracellular contents and participated in the pathogenesis of CD. They also found that GSDME converted TNF-α-induced IECs shedding into a pyroptotic cell death process, and GSDME depletion mice presented an alleviative intestinal barrier dysfunction (52).

Apart from the pyroptosis of IECs, pyroptosis in mucosal immune cells especially macrophages can also regulate intestinal inflammation strikingly. A recent study identified a dominant gain-of-function missense variant of NLRP3, encoded by rs772009059, promoted NLRP3 inflammasome activation and GSDMD-mediated pyroptosis in macrophages, thereby contributing to very-early onset IBD development (53). Cai et al. (54) observed that human umbilical cord mesenchymal stem cell (hucMSC)-derived exosomes carrying miR-378a-5p inhibited NLRP3/caspase-1/GSDMD pathway and abrogated macrophage pyroptosis to protect against DSS-induced colitis. *Roseburia intestinalis*, a butyrate−producing bacterium, -derived flagellin was demonstrated to inhibit the activation of NLRP3/caspase-1/GSDMD signaling-triggered pyroptosis in macrophages by targeting miR−223−3p and ameliorate colitis (55). In contrast, Ma et al. (56) showed that GSDMD and its mediated pyroptosis in macrophages protected against colitis by negatively regulating cyclic GMP-AMP synthase (cGAS)-dependent inflammation, while GSDMD deficiency in macrophages exacerbated experimental colitis.

**Figure 2:**
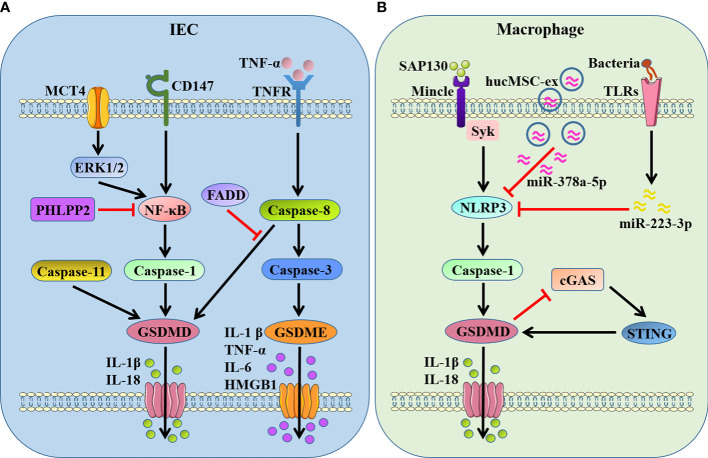
GSDMs-mediated pyroptosis in IBD-associated intestinal inflammation. **(A)** GSDMD- and GSDME-dependent pyroptosis in IECs. GSDMD is cleaved by active caspase-1/8/11 when NF-κB being activated by MCT4-ERK1/2 or CD147, promoting the release of IL-1β and IL-18. PHLPP2 and FADD inhibit GSDMD-dependent pyroptosis by suppressing NF-κB and caspase-8, respectively. GSDME is cleaved by active caspase-3 after caspase-8 activation in response to TNF-α stimulation, promoting the release of proinflammatory mediators IL-1β, TNF-α, IL-6 and HMGB1. **(B)** GSDMD-dependent pyroptosis in macrophages. GSDMD is cleaved by NLRP3/caspase-1 when Mincle/Syk pathway being activated in response to SAP130, and STING provokes GSDMD cleavage as well. In turn, GSDMD-mediated pyroptosis inhibits cGAS/STING signal. hucMSC-ex carrying miR-378a-5p and flagellin targeting miR−223−3p restrain NLRP3/caspase-1/GSDMD signaling-triggered pyroptosis. MCT4, monocarboxylate transporter 4; PHLPP2, pleckstrin homology domain leucine-rich repeat protein phosphatase 2; FADD, Fas associated with death domain; SAP130, spliceosome-associated protein 130; Mincle, macrophage-inducible C-type lectin; Syk, spleen tyrosine kinase; hucMSC-ex, human umbilical cord mesenchymal stem cell-derived exosomes; cGAS, cyclic GMP-AMP synthase; STING, stimulator of interferon genes.

Taken together, almost all the above studies support that GSDMD or GSDME-mediated pyroptosis in IECs or macrophages provoked intestinal inflammation and exacerbated the disease progression in IBD (**Figure 2**). This has stimulated the search for some drug interventions with the anti-pyroptosis effects to ameliorate intestinal inflammation. It is recently found that 10-hydroxy-2-decenoic acid (10-HDA), the most abundant fatty acid and major lipid component in royal jelly, alleviated DSS-induced colitis and enhancing colonic barrier function by regulating the NLRP3/caspase-1/GSDMD-mediated pyroptotic pathway (57). Schisandrin B, a type of natural products, was confirmed to suppress NLRP3/caspase-1/GSDMD-related pyroptosis in IECs of experimental colitis through the activation of AMP-activated protein kinase/Nuclear factor erythroid 2-related factor 2 (AMPK/Nrf2)-dependent reactive oxygen species (ROS)-induced mitochondrial damage (58). Furthermore, apple polyphenols extract (APE) has been reported to significantly ameliorate DSS-induced acute colitis through inhibiting caspase-1/caspase-11-GSDMD-dependent pyroptosis pathway in IECs (59).

### 4.2 Gasdermins are associated with endotoxemia- and sepsis‐related intestinal injury

Sepsis is defined as life-threatening organ dysfunction caused by a dysregulated host response to infection (60). The gut has long been considered to be the “motor” of sepsis, playing an essential role in the initiation and transmission of critical illness (61). A growing number of studies have confirmed GSDMs-mediated pyroptosis are involved in organ dysfunction of endotoxemia and sepsis, including in lung, kidney, liver and heart-related damage, but its role and specific mechanism in endotoxemia- or sepsis‐associated intestinal injury remains largely unknown.

Mandal et al. (62) investigated that neither caspase-11 nor pro-apoptotic caspase-8 was individually sufficient for endotoxic shock. They identified both caspase-11-GSDMD pyroptosis axis and caspase-8 apoptosis signaling collaborated to drive IECs shedding and amplify inflammatory signals associated with intestinal tissue damage during endotoxic shock (62). Intriguingly, another study indicated that both GSDMD-mediated pyroptosis and RIPK3-mediated necroptosis pathway contributed to inflammatory intestinal injury induced by bacterial sepsis (63). Sheng et al. (64) showed that intestinal colonization with commensal fungi inhibited GSDMD-mediated pyroptosis in response to lipopolysaccharide (LPS) challenge, while antifungal therapy aggravated endotoxin sepsis through promoting GSDMD cleavage in the distal small intestine. Moreover, CD4-positive T lymphocytes and IECs could secrete GSDMD-NT in high-fat diet (HFD)-induced systemic endotoxemia, and GSDMD-NT killed the *Proteobacteria phylum via* directly interacting with cardiolipin, thereby restraining gut barrier impairment and intestinal inflammation (65). Bromodomain-containing protein 4 (BRD4) was recently found to play a critical role in endotoxemia colon by regulating NLRP3/caspase-1/GSDMD-mediated pyroptosis, and BRD4 inhibition could prevent endotoxemia-induced colon damage through blocking inflammation-related pyroptosis (66).

Therefore, GSDMs-dependent pyroptosis is indeed involved in the progression of sepsis-related intestinal injury. Moderate pyroptosis in endotoxemia or sepsis can control pathogen infection, but excessive pyroptosis may lead to a dysregulated host immune response and even organ dysfunction. More studies are needed to elucidate the precise mechanism of activation and regulation of pyroptosis in intestinal injury associated with endotoxemia and sepsis.

### 4.3 Gasdermins-mediated lytic cell death in enteropathogenic microbial infections

The gastrointestinal tract not only has the functions of digestion and nutrient absorption, but also provides a physical barrier against amounts of commensal or pathogenic microbes in the gut lumen (67). The intestine employs multiple innate immune mechanisms to prevent and clear enteropathogenic microbial infections. Recent research upon GSDMs-dependent pyroptosis provided vital insights into host defense against pathogen.

*Salmonella*, a gram-negative facultative intracellular bacterium, is spread through polluted food or water and cause gastrointestinal disease that characterized by nausea, vomiting, abdominal pain and diarrhea after infection (68). *Salmonella* plasmid virulence C (SpvC) suppressed GSDMD-mediated pyroptosis in macrophages through negative modulation of both canonical and non-canonical inflammasome pathways, which related to the inhibition of intestinal inflammation while promotion of systemic bacterial dissemination (69). Ventayol et al. (70) demonstrated that *Salmonella* infection triggered NAIP/NLRC4/caspase-1/GSDMD-dependent focal epithelial contractions that preceded and uncoupled from IECs death and expulsion, thereby preventing subsequent tissue disintegration. Intestinal macrophages could also suffer NLRC4/caspase-1/GSDMD-dependent pyroptosis, which promoted inflammatory response and aggravated *Salmonella*-induced intestinal injury (71). Moreover, *Salmonella* was recently shown to induce pyroptosis of enteroendocrine L cells *via* NLRP3/caspase-1/GSDMD axis, which reduced the secretion of glucagon-like peptide 1, a significant intestinal hormone for regulating glucose homeostasis, and eventually resulted in hyperglycemia (72).

*Cronobacter sakazakii*, a gram-negative bacterial pathogen, was shown to upregulate NF-κB *via* TLR4/MyD88 to promote activation of the NLRP3 inflammasome and GSDMD-mediated pyroptosis, leading to the intestinal damage and development of necrotizing enterocolitis (73, 74). The translocated intimin receptor of enteropathogenic *Escherichia coli* triggered rapid Ca^2+^ influx in IECs, which induced LPS internalisation, resulting in the activation of caspase-4/GSDMD-driven pyroptosis of IECs (75). Melhem et al. (76) found that epithelial G protein-coupled receptor 35 protected against *Citrobacter rodentium* infection through guarding goblet cells from dysregulated caspase-11/GSDMD-mediated pyroptosis and maintaining mucosal barrier integrity. The canonical NLRP3/caspase-1/GSDMD pathway-dependent pyroptosis in macrophages contributed to the immunopathology and innate inflammatory responses upon gastrointestinal norovirus infection (77). Remarkably, Hansen et al. (78) recently elucidated that GSDMB, as a central executioner of intracellular bacterial killing, functioned lytic microbiocidal activity through recognition of phospholipids on gram-negative bacterial membranes. They also identified *Shigella flexneri* counteracted this host defense mechanism *via* ubiquitinated proteasome destruction pathway of GSDMB (78).

Intestinal parasite is another enteropathogenic microorganism that causes severe gastrointestinal inflammation and systemic infection. Enterocyte-intrinsic NLRP6/caspase-1/GSDMD-mediated pyroptosis played a critical role in enabling early control of *Cryptosporidium* replication by the local release of the proinflammatory cytokine IL-18 (79). Interestingly, *Entamoeba histolytica*-triggered caspase-1/4 activation in the cleavage of GSDMD to induce pore formation and sustain bioactive IL-1β secretion in the absence of pyroptotic cell death in macrophages (80). Type 2 cytokines, IL-4 or IL-13, triggered by worm infection, drastically up-regulated the expression of GSDMC in the gut, thereby enhancing the release of antiparasitic factors from enterocytes by GSDMC-mediated pyroptosis and facilitating the clearance of worms (81). Moreover, it was reported that epithelial O-linked N-Acetylglucosamine protein modification was initiated upon helminth infection, boosting GSDMC-mediated membrane pore formation and the unconventional secretion of IL-33, which contributed greatly to anti-helminth immunity and induced intestinal inflammation (23).

### 4.4 Gasdermins-regulated pyroptotic cell death during colorectal cancer

#### 4.4.1 Anti‐tumorigenesis effect of Gasdermins

**Figure 3:**
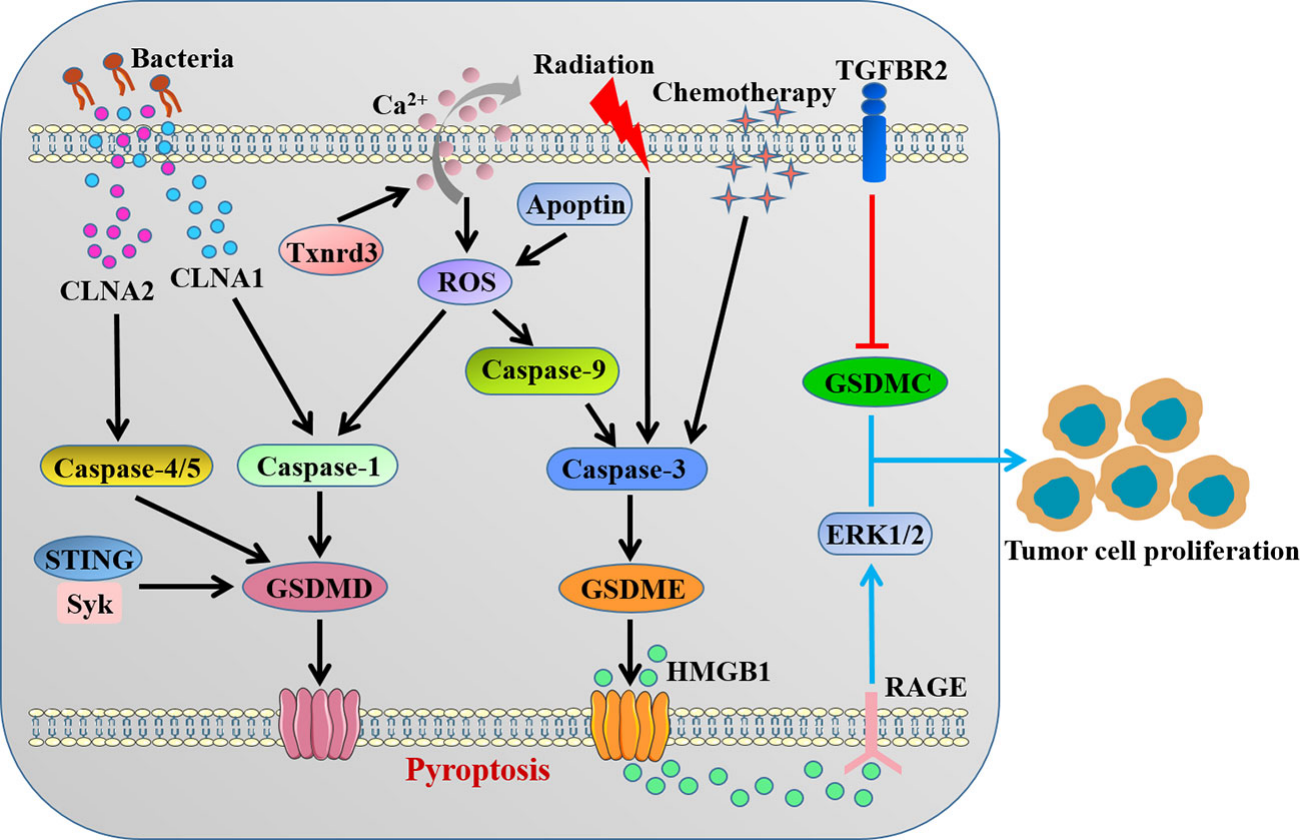
The contradictory role of GSDMs in colorectal cancer. CLNAs from probiotic bacterium and intracellular calcium outflow-caused ROS activate caspase-1/4/5 to cleave GSDMD. STING-mediated Syk signaling induces GSDMD-driven pyroptosis. Apoptin/ROS/caspase-9 axis, radiation and chemotherapy activate caspase-3 to cleave GSDME. The cleavage of GSDMD or GSDME inhibits the occurrence and progression of tumors by inducing pyroptosis. GSDMC and GSDME-related HMGB1 release promote tumor cell proliferation. CLNA, conjugated linolenic acid; Txnrd3, thioredoxin reductase 3; ROS, reactive oxygen species.

Accumulating evidence suggested that GSDMs-mediated pyroptosis might impact all stages of tumorigenesis and played a complex role in cancer biology depending on the different cellular context and genetic backgrounds (**Figure 3**). Obviously, as a type of cell death, pyroptosis inhibits the occurrence and progression of tumors. Our recent study clarified that STING-mediated Syk signaling attenuated the tumorigenesis of colitis-associated colorectal cancer (CAC) by enhancing GSDMD-driven pyroptosis of tumor cells (46). Thioredoxin reductase 3 (Txnrd3) as selenoprotein was recently proved to cause intracellular calcium outflow and increase oxidative stress in colonic epithelial cell line, thereby activating the canonical GSDMD-dependent pyroptosis to inhibit the growth and proliferation of colon cancer cells (82). Interestingly, it was reported that conjugated linolenic acid produced by the probiotic bacterium *Lactobacillus* exerted the anti-cancer effect through inducing GSDMD-mediated pyroptosis of colon cancer cells (83).

Previous studies indicated that *GSDME* gene methylation might play a vital role in the development of CRC, and *GSDME* as well as its methylation could be a promising molecular biomarker in CRC (84, 85). GSDME, rather than GSDMD, was demonstrated to be cleaved in lobaplatin-induced pyroptosis in colon cancer cells due to caspase-3 activation, which clarified the mechanism of lobaplatin eradicating neoplastic cells (86). It was reported that apoptin triggered caspase-9/caspase-3/GSDME-dependent pyroptosis of colon cancer cells through mitochondrial related ROS pathway and significantly inhibited tumor growth *in vivo* (87). Tan et al. (88) observed that radiation could induce caspase-3/GSDME-mediated pyroptosis in colon cancer cells, which recruited NK cells to provoke antitumor immunity and facilitated the radiosensitivity. The combination of oxaliplatin and farnesoid X receptor agonist GW4064 triggered caspase-3/GSDME-driven pyroptosis and slowed tumor growth, thereby enhancing the chemosensitivity of CRC (89). A recent study identified that small molecule inhibitors BI 2536 and (S)-(+)-camptothecin *via* organoid-based drug screen were able to induce GSDME-mediated pyroptosis concurrent with caspase-3-dependent apoptosis, which suppressed CRC tumor growth and enhanced the efficacy of immunotherapy (90). In contrast, Croes et al. (91) showed that there was no major differences for tumor features between GSDME^-/-^ and wild-type mice in both chemically induced and genetic intestinal cancer models, in spite of less severe inflammation observed in GSDME deficiency mice.

#### 4.4.2 The pro-tumor effect of Gasdermins

Beyond the anti-tumor function, the inflammatory microenvironment caused by pyroptosis may be suitable for tumor cell growth, which is closely related to the tumorigenesis. Recent evidence implicated that GSDMs also acted as oncogenes that participated in the initiation and progression of cancer. GSDMB was found to exert pro-tumor function by promoting proliferation, migration and invasion of tumor cells in both breast neoplasias and bladder cancer (92, 93). GSDMD could enhance tumor proliferation in non−small cell lung cancer and promote the invasion capacity of adenoid cystic carcinoma (94, 95).

For the colorectal cancer, Miguchi et al. (96) showed that GSDMC promoted tumor cell proliferation in colorectal carcinogenesis, and GSDMC facilitated xenograft tumor growth *in vivo*. In addition, GSDME-mediated pyroptosis promoted the development of CAC by releasing high-mobility group box protein 1 (HMGB1), which boosted tumor cell proliferation through the ERK1/2 pathway (97). Nevertheless, potential molecular mechanisms of pro-tumor effect of GSDMs in CRC remains largely unknown, further studies are warranted to unveil the relationship between GSDMs and tumor microenvironment.

### 4.5 Gasdermins-involved pyroptosis in other gut disorders

Intestinal I/R injury induced intestinal inflammation through activation of the NLRP3/caspase-1/GSDMD-driven pyroptosis, and metformin and dexmedetomidine were demonstrated to protect against intestine I/R injury by reducing expression of pyroptosis-related proteins (98, 99). Pyroptosis was recently observed to play an essential role in radiation-induced enteropathy, one of the most common and fatal complications of abdominal radiotherapy, and micheliolide significantly ameliorated radiation-induced intestinal tissue damage by lessening NLRP3/caspase-1/GSDMD pyroptosis pathway (100). Ye et al. (101) showed that Western diet induced increased myenteric neuronal pyroptosis *via* caspase-11/GSDMD pathway, leading to the myenteric nitrergic neuronal degeneration and colonic dysmotility. Caspase-11/GSDMD-triggered pyroptosis also contributed to acute graft-versus-host disease-related intestinal injury in allogeneic hematopoietic stem cell transplantation, while deletion of caspase-11 or GSDMD relieved intestinal inflammation (102). Circulating plasma exosomes derived from intestinal Behçet’s syndrome patients were proved to induce pyroptosis of intestinal epithelial cells *via* the activation of NLRP3/caspase-1/GSDMD pathway (103). Kerr et al. (104) revealed that GSDMD-mediated pyroptosis of intestinal macrophages resulted in increased gut permeability after thrombotic stroke, which provided a novel possible mechanism underlying gut complications after stroke. Further investigation was needed to clarify the pathogenic mechanism by which GSDMs facilitates gut homeostasis.

## 5 Pyroptosis-related PANoptosis in gut

It is widely known that GSDMs-dependent pyroptosis is a kind of inflammatory programmed cell death in response to pathogens or sterile insults. Emerging evidence states that multiple cell death pathways apart from pyroptosis, such as apoptosis and necroptosis, are induced during inflammation and immune response. Mechanistically, apoptosis is activated by the executioner caspase-3, caspase-6 or caspase-7, downstream of the initiator caspase-8, caspase-9 and caspase-10 (105). Necroptosis is initiated by phosphorylation of mixed lineage kinase domain-like pseudokinase (MLKL) downstream of the RIPK1 and RIPK3 signaling axis (105). Indeed, under such condition of homeostasis disbalance, a significant crosstalk among these three cell death pathways was observed, conceptualizing as a novel and united modality of death, namely PANoptosis (Pyroptosis-Apoptosis-Necroptosis) (106). PANoptosis is modulated by the multi-protein PANoptosome complex, which provides a molecular scaffold for simultaneous engagement of key molecules from pyroptosis, apoptosis and necroptosis (107). According to the biological functions, compositions of the PANoptosome consist of sensors, adaptors and catalytic effectors in general, such as Z-DNA binding protein 1 (ZBP1), FADD and caspase-1, respectively (108). Since PANoptosis has been triggered during various pathogenic infections, autoinflammatory diseases and cancer (107), indicating that it has significant pathophysiological relevance.

Recent studies suggested that interventions targeting PANoptosis-related molecules made a significant sense to the initiation and progression of gut diseases. Kanneganti’s group identified interferon regulatory factor 1 (IRF1) in both the myeloid and epithelial compartments could act as a master regulator of PANoptosis in the colon to induce cancer cell death, thus protecting against tumorigenesis in CAC (109). They confirmed synergism of cytokines TNF-α and IFN-γ was capable of inducing PANoptosis of colon cancer cells through activation of GSDMD, GSDME, caspase-8, caspase-3, caspase-7 and MLKL, thereby suppressing tumor growth (110). Their group also demonstrated that combining interferons and nuclear export inhibitors activated ZBP1-dependent PANoptosis and thus dramatically regressed colon tumor *in vivo* (111). By contrast, adenosine deaminase acting on RNA 1 (ADAR1), a RNA editor that critical for development and survival, interacted with ZBP1 and suppressed ZBP1-mediated PANoptosis, promoting tumorigenesis (111). Lin et al. (112) observed that cysteine desulfurase (NFS1), a rate-limiting enzyme in iron-sulfur cluster biogenesis, weakened oxaliplatin-based chemosensitivity of CRC by reducing the level of ROS to prevent PANoptosis.

**Figure 4:**
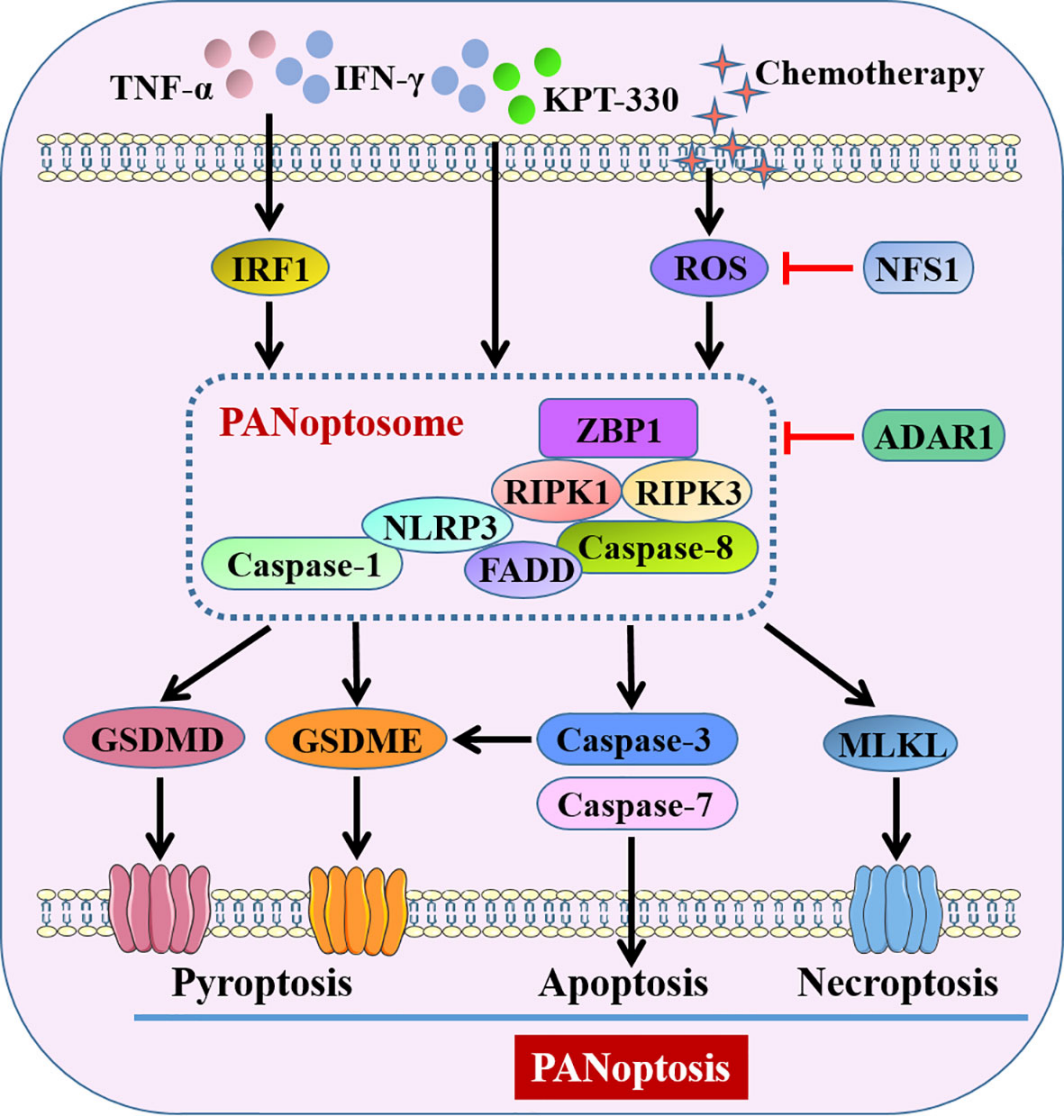
Pyroptosis-related PANoptosis in intestine. IFN-γ combined with TNF-α or KPT-330, and chemotherapy-induced ROS activate PANoptosome to trigger GSDMs-mediated pyroptosis, caspase-3/7-mediated apoptosis and MLKL-mediated necroptosis, conceptualizing as PANoptosis. ADAR1 and decreased ROS by NFS1 suppress PANoptosis through intervention of PANoptosome. KPT-330, a nuclear export inhibitor; NFS1, cysteine desulfurase; ADAR1, adenosine deaminase acting on RNA 1; ZBP1, Z-DNA binding protein 1; RIPK, receptor-interacting protein kinase; MLKL, mixed lineage kinase domain-like pseudokinase.

Therefore, the existing research results unanimously suggested induction of PANoptosis in cancer cells would be a promising strategy for inhibiting tumorigenesis of CRC (**Figure 4**). Further studies may be warranted to develop novel agonists that can drive cancer cell PANoptosis. In addition, more studies are urgently needed to understand the specific functions and mechanisms of PANoptosis in other gut disorders.

## 6 Intrinsic functions of Gasdermins in intestine independent of pyroptosis

**Figure 5:**
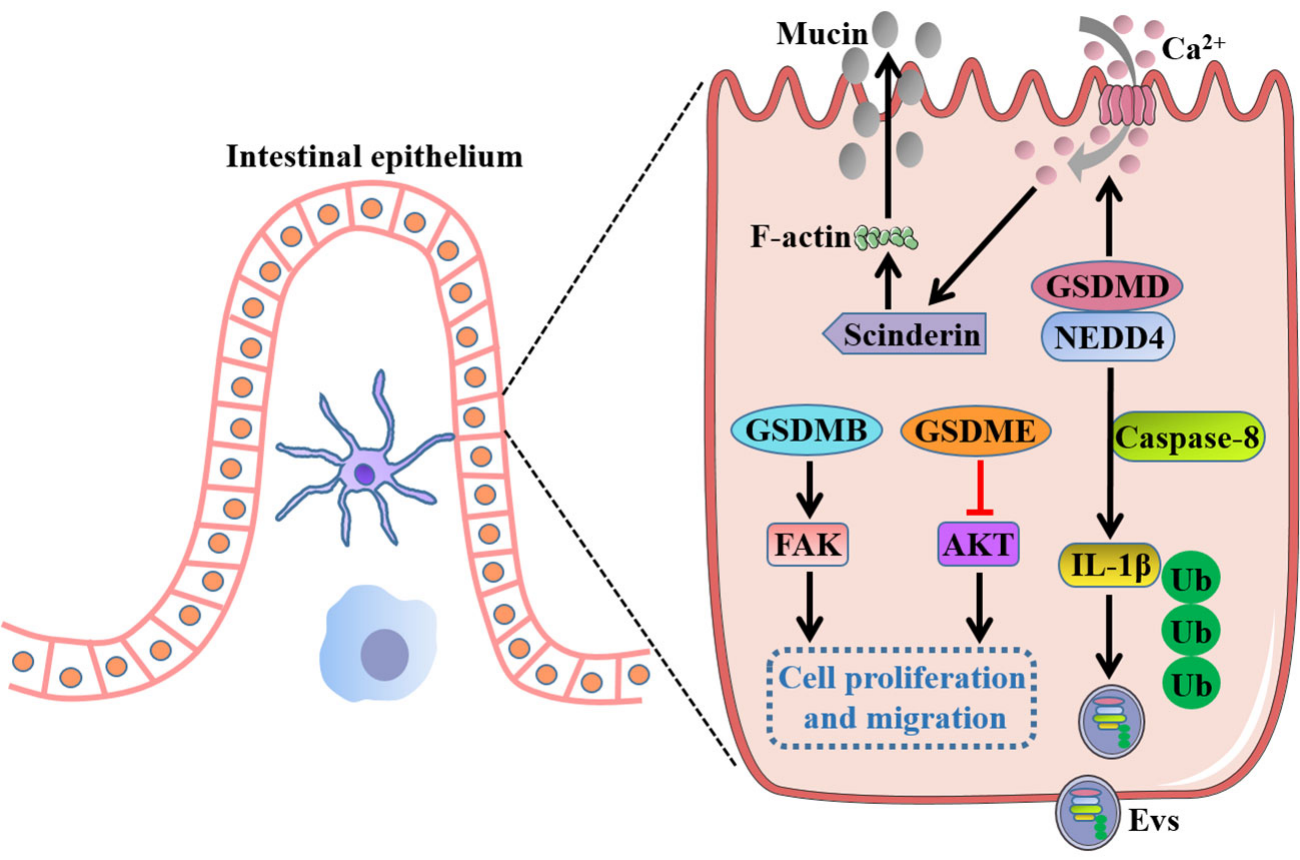
Intrinsic functions of GSDMs in gut independent of pyroptosis. Full-length GSDMB promotes cell proliferation and migration by regulation of FAK, whereas full-length GSDME suppresses cell growth by negative regulation of AKT pathway. GSDMD drives mucin secretion through calcium ion influx-dependent scinderin-mediated cortical F-actin disassembly, and recruits NEDD4 to mediate the release of polyubiquitinated IL-1β in the form of Evs *via* a caspase-8-dependent manner. FAK, focal adhesion kinase; NEDD4, an E3 ligase; Evs, extracellular vesicles.

The GSDMs have been primarily known for their role as pore-forming effector proteins upon cleavage, oligomerization, and translocation of the N-terminal domain to the plasma membrane to cause pyroptosis. Nonetheless, GSDMs are also expressed in many healthy tissues without obvious pyroptosis and inflammation especially the gastrointestinal tract, suggesting potential physiological functions associated with GSDMs that independent of pyroptosis may be modulated in the steady state. Recently, emerging studies have confirmed the nonpyroptotic role of GSDMs during maintenance of intestinal homeostasis (**Figure 5**).

Rana et al. (20) reported GSDMB was increased in inflamed colonocytes and crypt top colonocytes of IBD patients, and GSDMB enhanced IECs proliferation and migration by regulation of focal adhesion kinase (FAK) phosphorylation rather than its driven pyroptosis, thus promoting restoration of epithelial barrier function and the resolution of inflammation. GSDMD was recently found to recruit the E3 ligase NEDD4 to mediate the release of polyubiquitinated IL-1β *via* a caspase-8-dependent, nonpyroptotic pathway in IECs, which was crucial for the development of intestinal inflammation (113). Moreover, a recent study showed that GSDMD drove mucin secretion through calcium ion-dependent scinderin-mediated cortical F-actin disassembly, a key step in granule exocytosis, highlighting the distinctive function of GSDMD in intestinal goblet cell mucin secretion and mucus layer formation that uncoupled from pyroptotic cell death (114). Interestingly, a previous study showed that there were no gross histological abnormalities in the small intestinal or the colonic epithelium of GSDMD^-/-^ mice, and GSDMD^-/-^ mice appeared to exhibit comparable numbers of the differentiated cell lineages with wild-type mice such as goblet cells, Paneth cells and enteroendocrine cells, indicating an inessential role of GSDMD for intestinal epithelium development (115). These inconsistent results for GSDMD in gut homeostasis may attract researchers to further investigate the inherent functions of GSDMs. In addition, full-length GSDME was demonstrated to markedly decrease the cell growth and colony-forming ability of colon cancer cells by negative regulation of AKT pathway, whereas knockdown of GSDME enhanced cancer cell cycle progression and cell growth, implicating a potential tumor suppressor role for GSDME in CRC (116). However, the precise mechanism by which GSDMs exert the intrinsic functions independent of pyroptosis in intestinal homeostasis remains largely unknown and needs to be further elucidated.

## 7 Conclusions and perspectives

GSDMs are widely expressed in the intestine, while GSDMA, GSDMB, GSDMC and GSDME tend to appear prominently in intestinal epithelium, and GSDMD presents in both IECs and lamina propria immune cells. There is no doubt that GSDMs play a vital role in the intestinal mucosal immunity and homeostasis, due to its mediated pyroptosis, related PANoptosis, and inherent functions independent of pyroptotic cell death. The promotion or resistance effects of GSDMs on intestinal inflammation and tumorigenesis may depend on the biological context, in particular the interaction of GSDMs-expressing cells with the immune or tumor microenvironment. In these sense, the development of both agonists and blockers of GSDMs might be useful to govern therapy response and disease progression.

In fact, current research on GSDMs is just the tip of the iceberg, there are still multiple hurdles to resolve to fully unveil the role of GSDMs in gut homeostasis. Firstly, active GSDMs are important during cell death and inflammation, and GSDMs have been found to be cleaved and activated by caspases, SpeB virulence factor, neutrophil elastase, cathepsin G or granzymes. Whether GSDMs can be cleaved and activated by additional cellular proteases as well as pathogen-encoded proteases in gut remains to be explored. Secondly, as the key perpetrators of inflammation, the specific mechanisms of GSDMs being initiated and activated by which stimulating factors in intestinal homeostasis need to be further determined. Thirdly, it is notable that GSDMs are aberrantly expressed in CRC and are observed to be either promoters or suppressers of cancer. However, how GSDMs function to shape CRC cells or the tumor microenvironment in pyroptosis-dependent or -independent manners are largely unclear. Fourthly, several mutations of GSDMs have been demonstrated to increase the incidence risk of certain gut diseases, but whether it is caused by pyroptotic cell death or inherent function of GSDMs needs future investigation. In any case further insights into the roles of GSDMs in gut will not only strengthen our understanding of the daedal mechanisms underlying intestinal inflammation and tumorigenesis but will also help to exploit novel therapeutic strategies for gut homeostasis imbalance.

## Author contributions

WG and KY drafted the manuscript and designed the figure. WZ and JZ revised the manuscript. JY, KG and XS conceived the topic. All authors contributed to the article and approved the submitted version.

## Funding

This work was supported by the Institutional Foundation of The First Affiliated Hospital of Xi'an Jiaotong University (Grant serial numbers: 2022QN-07) and National Natural Science Foundation of China (Grant serial numbers: 82002547).

## Conflict of interest

The authors declare that the research was conducted in the absence of any commercial or financial relationships that could be construed as a potential conflict of interest.

## Publisher’s note

All claims expressed in this article are solely those of the authors and do not necessarily represent those of their affiliated organizations, or those of the publisher, the editors and the reviewers. Any product that may be evaluated in this article, or claim that may be made by its manufacturer, is not guaranteed or endorsed by the publisher.
